# The heme oxygenase-1 and c-FLIP in acute myeloid leukemias: two non-redundant but mutually exclusive cellular safeguards protecting cells against TNF-induced cell death?

**DOI:** 10.18632/oncotarget.163

**Published:** 2010-09-16

**Authors:** S. Shirley, O. Micheau

**Affiliations:** ^1^INSERM, U866, Dijon, F-21079 France; Faculty of Medicine and Pharmacy, Univ. Bourgogne, Dijon, F-21079, France; ^2^Centre Georges-François Leclerc, Dijon, F-21000, France

**Figure 1:**
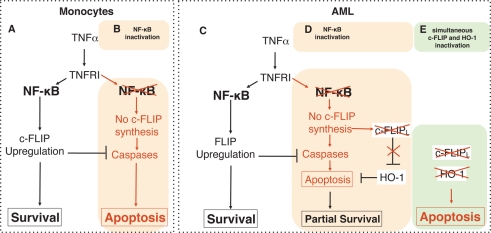
Contribution of HO-1 and c-FLIPL to the regulation of TNF signalling in monocytes and acute myeloid leukemia cells (AML) (A) In monocytes, engagement of TNFR1 by TNFα induces activation of NF-κB, leading to up-regulation of FLIP and inhibition of cell death, however inactivation of NF-κB (B) prevents FLIP neosynthesis, allowing caspase activation and apoptosis. (C) AML cells are resistant to TNFα-induced apoptosis, even upon inactivation of NF- κB (D), due to the up-regulation of HO-1. (E) Simultaneous inactivation of c-FLIP and HO-1 enhances TNF-induced cell death.

TNF-induced apoptosis is tightly regulated by the NF-κB pathway. Under physiologic conditions, TNFα stimulation induces NF-κB activation and cell survival, due to the regulation of anti-apoptotic genes, including c-FLIP, a caspase-8 inhibitor, whose expression is sufficient to protect cells against TNF-induced apoptosis. TNF triggers cell death only in circumstances where the NF-κB pathway is defective. Rushworth and collaborators have recently demonstrated, however, that the heme oxygenase-1 (HO-1), also known as Heat shock protein 32 (Hsp32) [1], like c-FLIP, can afford protection against TNF-induced cell death in AML cells, despite NF-κB inactivation [2]. They now provide evidence that TNF mediated HO-1 up-regulation, is negatively regulated by c-FLIP, revealing a novel negative regulatory feedback loop controlling apoptosis induced by TNRI (Figure 1).

In contrast to Fas or TRAIL receptor-mediated cell death, apoptosis induced by TNFRI is a two-step process that requires the formation of two sequential signalling complexes [3]. The plasma membrane-bound complex I, including TNFR1, TRADD, RIP1 and TRAF2, is dedicated to the activation of the survival pathway NF-κB. FADD and caspase-8 are recruited in the “cytosolic” complex, also coined complex II, which is devoid of TNFRI, triggering caspase-8 activation and apoptosis [3]. In the vast majority of cells, however, activation of NF-κB induces protection against TNF-induced cell death [4]. Several anti-apoptotic genes are regulated by NF-κB [5], but so far only c-FLIP has been demonstrated to afford full protection when expressed alone [6,7]. Activation of complex II and thus triggering of the apoptotic program is generally thought to occur in NF-κB defective cells due to the lack of c-FLIP supply [8].

HO-1 is a stress-related anti-apoptotic molecule that has been implicated in enhanced survival of cancer cells and in drug-resistance [1]. Overexpression of HO-1 protects cells from H2O2-, Fas- or TNF-induced apoptosis [9-11]. Unlike HO-2, the second evolutionary conserved heme oxygenase isoenzyme, HO-1 is not expressed constitutively. HO-1 is generally induced under oxidative stress enabling enhanced free heme catabolism and inhibition of programmed cell death[1]. HO-1 mediated cytoprotection has been assigned to the heme catabolism sub-product Fe2+, which triggers reactive oxygen species (ROS) production and NF-κB activation [12]. Induced expression of HO-1 by IL-1 and TNFα was suggested to involve protein kinase c, calcium and phospholipase A2 [13]. Activation of the PkB/Akt pathway and induction of Nrf2 were shown to induce HO-1 up-regulation upon H2O2 stimulation[9]. More recently it was shown that TNF-mediated ROS production, in NF-κB inactivated AML cells, induced the activation of the transcription factor Nrf2 leading to HO-1 up-regulation [2]. The cytoprotective activity of HO-1 in endothelial cells was demonstrated to require NF-κB activation by TNFα [14]. Interestingly, HO-1-mediated inhibition of TNFRI-induced apoptosis, in NF-κB defective cells, can be restored by the ectopic expression of some NF-κB regulated genes such as c-IAP2, A1 or A20 [14]. Furthermore, HO-1-mediated protection against TNF-induced cell death is not restricted to tumour cells, as endothelial cells or human fibroblasts induced to express HO-1 fail to undergo apoptosis [14,15].

Remarkably, and in contrast to most studies demonstrating that inhibition of the NF-κB pathway restores TNF-induced cell death in normal and cancer cells, Rushworth et al. demonstrate in this issue that NF-κB inhibition only affords partial restoration of apoptosis in AML cells, due to the up-regulation of HO-1. Accordingly, inactivation of c-FLIP_L_ expression was sufficient to trigger the accumulation of HO-1 in the absence of TNF, though apoptosis following TNFα stimulation was only partially restored. Accordingly, inactivation of c-FLIP_L_ expression in these cells, albeit partially restoring TNFα-induced apoptosis, in the absence of TNF, triggered the accumulation of HO-1. However, simultaneous inactivation of c-FLIP_L_ and HO-1 significantly enhanced AML cell sensitivity to TNFα. Rushworth et al. make the critical observation that induction of HO-1 expression is negatively regulated at the steady state by c-FLIP_L_, but not the short forms of c-FLIP, providing a plausible explanation for the resistance of AML cells to TNF-induced apoptosis, despite inactivation of the NF-κB pathway.

These results demonstrate that HO-1 exerts cytoprotection in AML cells, irrespective of NF-κB activation, and suggest in addition that HO-1 and c-FLIP_L_ may negatively regulate TNF-induced cell death in a non-redundant, but exclusive manner. Of particular interest, c-FLIP_L_ down-regulation was unable to promote HO-1 expression in monocytes. Thus the markedly increased expression of c-FLIP_L_ and the constitutive activation of NF-κB in erythroleukemia cells [16] would support the proposal that negative regulation of HO-1 expression by c-FLIP_L,_ at the basal level, might require sustained NF-κB activation. In line with this hypothesis, it has been demonstrated in the past that over-expression of c-FLIP, or at least its amino acid terminal portion, could induce NF-κB activation [17-20]. It is not clear, however, whether NF-κB activation alone is sufficient to repress HO-1. ROS production, through the activation of Nrf2, may also induce the restoration of HO-1 expression in cells in which c-FLIP_L_ has been inactivated, as c-FLIP down-regulation was shown to induce ROS production in some tumour cells [21], while its over-expression produces the opposite effect [22].

While it is clear that the molecular mechanisms underlying c-FLIP_L_-mediated HO-1 repression at the basal level needs to be explored more precisely, the possibility that HO-1 itself may regulate c-FLIP expression, through its ability to inhibit NF-κB activation, or to induce ROS remains an open question. In line with this hypothesis, it has recently been demonstrated that HO-1 was able to impair NF-κB nuclear translocation in cardiomyocytes [23] and that ROS production can trigger the degradation of c-FLIP in an ubiquitylation-dependent manner [24]. Mutual regulation of these cellular “safeguards” would thus certainly be beneficial for tumour cells to maintain a high level of protection against TNF-induced killing. Altogether these findings uncover a novel cell-decision regulatory mechanism controlling cell death signalling induced by TNFRI, which may extend to other death-inducing ligands of the TNF family.
